# Supplementary material for: Clonal Diversity of Candida auris, Candida blankii, and Kodamaea ohmeri Isolated from Septicemia and Otomycosis in Bangladesh as Determined by Multilocus Sequence Typing
Source: J Fungi (Basel). 2023 Jun 12;9(6):658. doi: 10.3390/jof9060658 (PMC10301082; doi:10.3390/jof9060658)
Supplement: Supplementary file 1 [file jof-09-00658-s001.zip › Table-S6-JoF-SathiR1.pdf]

Table S6 Sequence identity among alleles of ITS, *RPB1*, *RPB2*, and D1/D2 locus of *C. auris*, *C. blankii*, and *K. ohmeri*

ITS

	1	2	3	4	5	6
1: C.auris-ITS-3	100.00	98.94	97.06	95.72	97.61	97.34
2: C.auris-ITS-6	98.94	100.00	97.33	95.19	97.07	96.81
3: C.auris-ITS-2	97.06	97.33	100.00	96.79	98.67	98.40
4: C.auris-ITS-5	95.72	95.19	96.79	100.00	98.14	98.41
5: C.auris-ITS-1	97.61	97.07	98.67	98.14	100.00	99.74
6: C.auris-ITS-4	97.34	96.81	98.40	98.41	99.74	100.00

	1	2	3	4
1: C.blankii-ITS-4	100.00	95.92	97.51	97.28
2: C.blankii-ITS-2	95.92	100.00	97.75	97.52
3: C.blankii-ITS-1	97.51	97.75	100.00	99.78
4: C.blankii-ITS-3	97.28	97.52	99.78	100.00

	1	2	3	4	5	6	7	8
1: K.ohmeri-ITS-1	100.00	99.74	98.43	98.43	98.69	98.43	96.34	97.12
2: K.ohmeri-ITS-5	99.74	100.00	98.17	98.69	98.43	98.69	96.07	96.86
3: K.ohmeri-ITS-6	98.43	98.17	100.00	99.21	99.74	99.48	94.52	95.30
4: K.ohmeri-ITS-8	98.43	98.69	99.21	100.00	99.48	99.74	94.76	95.55
5: K.ohmeri-ITS-2	98.69	98.43	99.74	99.48	100.00	99.74	95.03	95.81
6: K.ohmeri-ITS-4	98.43	98.69	99.48	99.74	99.74	100.00	94.76	95.55
7: K.ohmeri-ITS-3	96.34	96.07	94.52	94.76	95.03	94.76	100.00	99.22
8: K.ohmeri-ITS-7	97.12	96.86	95.30	95.55	95.81	95.55	99.22	100.00

RPB1

	1	2	3
1: C.auris-RPB1-2	100.00	99.68	99.84
2: C.auris-RPB1-1	99.68	100.00	99.84
3: C.auris-RPB1-3	99.84	99.84	100.00

	1	2	3	4	5	6	7
1: K.ohmeri-RPB1-3	100.00	98.52	98.66	98.96	98.96	99.26	99.11
2: K.ohmeri-RPB1-5	98.52	100.00	99.85	99.55	99.55	99.26	99.41
3: K.ohmeri-RPB1-7	98.66	99.85	100.00	99.70	99.70	99.41	99.55
4: K.ohmeri-RPB1-2	98.96	99.55	99.70	100.00	99.41	99.70	99.55
5: K.ohmeri-RPB1-6	98.96	99.55	99.70	99.41	100.00	99.70	99.85
6: K.ohmeri-RPB1-1	99.26	99.26	99.41	99.70	99.70	100.00	99.85
7: K.ohmeri-RPB1-4	99.11	99.41	99.55	99.55	99.85	99.85	100.00

RPB2

	1	2	3	4
1: C.auris-RPB2-1	100.00	99.90	98.92	99.11
2: C.auris-RPB2-2	99.90	100.00	99.01	99.01
3: C.auris-RPB2-3	98.92	99.01	100.00	99.61
4: C.auris-RPB2-4	99.11	99.01	99.61	100.00

	1	2
1: C.blankii-RPB2-1	100.00	99.90
2: C.blankii-RPB2-2	99.90	100.00

	1	2	3	4	5	6	7	8
1: K.ohmeri-RPB2-8	100.00	99.39	99.39	98.99	98.89	98.89	98.99	98.69
2: K.ohmeri-RPB2-3	99.39	100.00	99.80	99.60	99.49	99.49	99.19	99.09
3: K.ohmeri-RPB2-4	99.39	99.80	100.00	99.39	99.29	99.29	98.99	98.89
4: K.ohmeri-RPB2-1	98.99	99.60	99.39	100.00	99.49	99.70	99.19	99.09
5: K.ohmeri-RPB2-6	98.89	99.49	99.29	99.49	100.00	99.80	99.29	99.19
6: K.ohmeri-RPB2-7	98.89	99.49	99.29	99.70	99.80	100.00	99.29	99.19
7: K.ohmeri-RPB2-2	98.99	99.19	98.99	99.19	99.29	99.29	100.00	99.29
8: K.ohmeri-RPB2-5	98.69	99.09	98.89	99.09	99.19	99.19	99.29	100.00

D1/D2

	1	2	3	4
1: C.auris-D1D2-2	100.00	99.26	97.21	96.64
2: C.auris-D1D2-3	99.26	100.00	96.46	96.64
3: C.auris-D1D2-1	97.21	96.46	100.00	99.07
4: C.auris-D1D2-4	96.64	96.64	99.07	100.00

	1	2
1: C.blankii-D1D2-1	100.00	99.66
2: C.blankii-D1D2-2	99.66	100.00

	1	2	3	4
1: K.ohmeri-D1D2-1	100.00	99.81	99.43	99.24
2: K.ohmeri-D1D2-2	99.81	100.00	99.24	99.05
3: K.ohmeri-D1D2-3	99.43	99.24	100.00	99.81
4: K.ohmeri-D1D2-4	99.24	99.05	99.81	100.00
